# Overcoming resistance to immune checkpoint inhibitors in hepatocellular carcinoma: Challenges and opportunities

**DOI:** 10.3389/fonc.2022.958720

**Published:** 2022-09-02

**Authors:** Qingqing Xie, Pengfei Zhang, Yuanyuan Wang, Wuxuan Mei, Changchun Zeng

**Affiliations:** ^1^ Department of Medical Laboratory, Shenzhen Longhua District Central Hospital, Guangdong Medical University, Shenzhen, China; ^2^ Xianning Medical College, Hubei University of Science and Technology, Xianning, China

**Keywords:** hepatocellular carcinoma, resistance, immune checkpoint inhibitors, mechanism, combination therapy

## Abstract

Hepatocellular carcinoma is one of the leading causes of cancer mortality globally, and its incidence is increasing. Immune checkpoint therapy has revolutionized the treatment of hepatocellular carcinoma over the past few years. However, only a limited proportion of patients with hepatocellular carcinoma respond to immunotherapy. Despite the significant breakthroughs, the molecular mechanisms that drive immune responses and evasion are largely unresolved. Predicting tumor response and resistance to immune checkpoint inhibitors is a significant challenge. In this review, we focus on the current research progress of immune checkpoint inhibitors in hepatocellular carcinoma. Importantly, this review highlights the underlying mechanisms of resistance to immune checkpoint inhibitors and summarizes potential strategies to overcome the resistance to immune checkpoint inhibitors in hepatocellular carcinoma.

## Introduction

With an estimated 906,000 new cases and 830,000 deaths in 2020, primary liver cancer is the sixth most often diagnosed cancer and the third leading cause of cancer mortality globally. Hepatocellular carcinoma (HCC) accounts for 75%–85% of all primary liver cancer cases. The overwhelming number of patients diagnosed with HCC is in advanced stages, and just a tiny number are candidates for potentially effective treatment (1). The multikinase inhibitors sorafenib and lenvatinib have been the systematic treatment of choice for individuals with advanced HCC until recently. In recent years, the rapid development of immune checkpoint inhibitors (ICIs), particularly those targeting cytotoxic T lymphocyte-associated antigen-4 (CTLA-4), programmed death-1 (PD-1), and programmed death ligand-1 (PD-L1), has altered therapeutic alternatives for solid malignancies. In HCC, ICIs have evolved as potentially promising anticancer therapeutic approaches, and several ICIs have been approved for the treatment of HCC, resulting in a paradigm shift in the management of HCC. In particular, the combination of atezolizumab and bevacizumab has emerged as the preferred systemic treatment option for patients with advanced or inoperable HCC in 2021 (2–5). Although ICIs offer new hope to patients with advanced HCC, ICI alone, such as nivolumab and pembrolizumab, is only moderately effective in patients with HCC. The findings of the current trial show significant heterogeneity in the extent to which tumors respond to these ICIs. In a phase 3 trial (CheckMate 459), nivolumab monotherapy did not significantly enhance overall survival (OS) in the first-line setting for advanced HCC patients compared with sorafenib. The negative results suggest that nivolumab monotherapy might be a first-line systemic therapy option for patients who are not candidates for tyrosine kinase inhibitors or antiangiogenic agents (6). Similarly, second-line pembrolizumab treatment did not significantly improve OS and progression-free survival (PFS) compared with sorafenib in a phase 3 trial (KEYNOTE-240) (7).

Potential predictors and biomarkers of ICIs in HCC with the ability to explain the heterogeneity would be beneficial for optimizing patient selection in the clinical setting. Although immunohistochemistry for PD-L1 is utilized to identify a variety of cancers that are most likely to respond to anti-PD-1/PD-L1 therapy, including bladder cancer, breast cancer, non-small cell lung cancer, head and neck cancer, gastric cancer, esophageal and esophagogastric junction cancer, cervical cancer, and vulvar cancer, its predictive value for HCC response is questionable (8). Multiple cancers with a high tumor mutational burden (TMB), such as bone cancer, melanoma, breast cancer, head and neck cancer, prostate cancer, and testicular cancer, are more likely to respond to ICIs. However, its ability to predict HCC response is debatable (9, 10). Furthermore, high microsatellite instability (MSI-H) or deficient mismatch repair (dMMR) may predict immunotherapy response in advanced solid tumors, including bone cancer, breast cancer, cervical cancer, colon cancer, esophageal and esophagogastric junction cancer, gastric cancer, head and neck cancer, extrahepatic cholangiocarcinoma, gallbladder cancer, intrahepatic cholangiocarcinoma, pancreatic adenocarcinoma, penile cancer, epithelial ovarian cancer/fallopian tube cancer/primary peritoneal cancer, prostate cancer, rectal cancer, small bowel adenocarcinoma, testicular cancer, thyroid carcinoma, uterine neoplasms, and vulvar cancer (11, 12). Dostarlimab-gxly (an anti-PD-1 antibody) can be considered in patients with recurrent or advanced HCC carrying MSI-H/dMMR (13, 14).

Although ICIs have revolutionized the management of HCC, the treatment outcomes may be unpredictable and inconsistent. A considerable proportion of patients do not respond to ICIs or develop resistance to them. Determining which patients can benefit from immunotherapy is one of the significant challenges. Combination therapy strategies may improve the efficacy of ICIs (15). This review sheds light on the mechanisms behind immunotherapy resistance in HCC and provides prospective options for overcoming ICI resistance.

## Immune checkpoint inhibitors in hepatocellular carcinoma

Cancer cells block T-cell activation and produce immune checkpoint proteins on T cells, making immune activation more challenging. When CTLA-4 and PD-1 bind to their ligands, T-cell activity is reduced, and antitumor immunity is further blocked. As a result, CTLA-4, PD-1, or PD-L1 inhibitors may be effective in cancer immunotherapies (**Table 1**) (16). CTLA-4 acts as a negative modulator of T-cell effector activity, making it an appealing target for cancer treatment. In the CTLA-4 pathway, exocytosis of CTLA-4 from intracellular vesicles to the T-cell surface is activated by binding signals of T-cell receptor (TCR)-major histocompatibility complex (MHC) and CD28-B7. CLTA-4 can bind to B7 with a higher binding affinity than CD28, resulting in the decreased activity of T cells. CTLA-4 inhibitors can interfere with the interaction of CTLA-4 on T cells with B7 ligands on antigen-presenting cells (APCs), suppressing regulatory T cell (Treg)-related immunosuppression and promoting the function of T-cell effector, resulting in an immune response. The T-cell effector function is suppressed by the interaction between PD-1 on T cells and its ligand PD-L1 on APCs. The PD-1/PD-L1 pathway is a negative modulator of immune responses and a crucial route for tumor immune escape. PD-1 inhibitors specifically target PD-1 and block the interaction between PD-L1 and PD-1. Inhibitors that target PD-L1 prevent PD-L1 from binding to PD-1. PD-1/PD-L1 inhibitors disrupt the feedback loop between T cells and tumor cells in the tumor microenvironment (TME), restoring T-cell effector function and increasing antitumor efficacy (17).

**Table 1:** Selected trials of immune checkpoint inhibitors in advanced hepatocellular carcinoma.

Trial Name	Phase	Intervention	Target	Setting
NCT02576509	Phase III	Nivolumab	PD-1	Advanced HCC
NCT02702401	Phase III	Pembrolizumab	PD-1	Participants with previously systemically treated advanced HCC
NCT03434379	Phase III	Bevacizumab + Atezolizumab	VEGF; PD-L1	Untreated locally advanced or metastatic HCC
NCT04039607	Phase III	Nivolumab + Ipilimumab	PD-1; CTLA-4	Advanced HCC
NCT03764293	Phase III	Camrelizumab + Apatinib	PD-1; VEGF	First-line therapy in patients with locally advanced or metastatic and unresectable HCC
NCT03755791	Phase III	Atezolizumab + Cabozantinib	VEGF; PD-L1	Advanced HCC who have not received previous systemic anticancer therapy
NCT03298451	Phase III	Durvalumab + Tremelimumab	PD-1; CTLA-4	Advanced HCC
NCT03713593	Phase III	Lenvatinib + Pembrolizumab	VEGF; PD-1	First-line therapy in participants with advanced HCC
NCT03794440	Phase II/III	Sintilimab + IBI308	PD-1; VEGF	Advanced HCC
NCT03412773	Phase III	Tislelizumab	PD-1	Unresectable HCC
NCT04102098	Phase III	Bevacizumab + Atezolizumab	VEGF; PD-L1	Adjuvant therapy in patients with HCC at high risk of recurrence after surgical resection or ablation
NCT04665609	Phase III	Thermal Ablation+Anlotinib+TQB2450	VEGF; PD-L1	Advanced HCC
NCT04167293	Phase II/III	Radiation + Sintilimab	PD-1	HCC
NCT04709380	Phase III	Radiotherapy + Toripalimab	PD-1	Advanced HCC with portal vein/hepatic vein tumor thrombosis
NCT04523493	Phase III	Toripalimab + Lenvatinib	PD-1; VEGF	Advanced HCC
NCT04803994	Phase III	Bevacizumab + Atezolizumab	VEGF; PD-L1	Intermediate-stage HCC
NCT03867084	Phase III	Pembrolizumab	PD-1	Adjuvant therapy in participants with HCC and complete radiological response after surgical resection or local ablation
NCT04246177	Phase III	Lenvatinib + Pembrolizumab + TACE	VEGF; PD-1	Incurable/non-metastatic HCC
NCT03062358	Phase III	Pembrolizumab	PD-1	Previously treated advanced HCC
NCT04044651	Phase III	Lenvatinib + Nivolumab	VEGF; PD-1	Advanced HCC with hepatitis B virus infection
NCT03605706	Phase III	Camrelizumab + FOLFOX4	PD-1	Advanced HCC who have never received prior systemic treatment
NCT04465734	Phase III	HLX10 + HLX04	PD-1; VEGF	First-line treatment in patients with locally advanced or metastatic HCC
NCT03859128	Phase II/III	Toripalimab	PD-1	Adjuvant therapy in HCC after radical resection
NCT05250843	Phase II/III	TACE/HAIC + Lenvatinib + Sintilimab	VEGF; PD-1	Neoadjuvant therapy for intermediate-stage HCC

HAIC, hepatic arterial infusion of FOLFOX-based chemotherapy; TACE, transarterial chemoembolization; HCC, hepatocellular carcinoma.

Patients with advanced HCC receiving 3 mg/kg nivolumab (an anti-PD-1 antibody) exhibited an objective response rate of 20% (95% CI: 15–26) in a phase I/II trial (CheckMate-040), contributing to its approval as the first second-line systemic treatment for HCC. In this trial, 25% (12/48) of patients had grade 3/4 treatment-related adverse events. Treatment-related serious adverse events occurred in 6% (3/50) of patients, including adrenal insufficiency, liver disorder, and pemphigoid (2).

Pembrolizumab (an anti-PD-1 antibody) also received accelerated approval as a second-line treatment after reporting an objective response in 17% (18/104) of advanced HCC patients in a phase II trial (KEYNOTE-224). In this trial, 28% (29/104) of patients had grade 3/4 treatment-related adverse events, mainly including increased aspartate aminotransferase (7%), fatigue (4%), increased alanine aminotransferase (4%), hyperbilirubinemia (2%), and adrenal insufficiency (2%) (3). In an Asian subgroup analysis of the KEYNOTE-240 trial, the pembrolizumab group had a median PFS of 2.8 months (95% CI: 2.6–4.1), whereas the placebo group had a median OS of 1.4 months (95% CI: 1.4–2.4) [hazard ratio (HR): 0.48; 95% CI: 0.32–0.70] after a median follow-up of 13.8 months for the pembrolizumab group and 8.3 months for the placebo group. The median OS was 13.8 months (95% CI: 10.1–16.9) and 8.3 months (95% CI: 6.3–11.8) (HR: 0.55; 95% CI: 0.37–0.80) for pembrolizumab and placebo, respectively. In this trial, 58.9% (63/107) and 48.0% (24/50) of patients in the pembrolizumab and placebo groups experienced treatment-related adverse events, respectively. Grade 3–5 treatment-related adverse events occurred in 13.1% (14/107) and 4.0% (2/50) of patients in the pembrolizumab and placebo groups (18).

The phase I/II trial (CheckMate 040) was the first to exhibit the efficacy of a CTLA-4 inhibitor (ipilimumab) in combination with a PD-1 inhibitor (nivolumab) in patients with advanced HCC. Patients receiving ipilimumab (3 mg/kg) plus nivolumab (1 mg/kg) every 3 weeks (four doses) followed by nivolumab 240 mg every 2 weeks achieved an investigator-assessed objective response rate of 32%. One grade 5 treatment-related adverse event of pneumonitis occurred (4). Furthermore, the IMbrave150 trial demonstrated that the combination of atezolizumab (an anti-PD-L1 antibody) and bevacizumab [an anti- vascular endothelial growth factor A (anti- VEGFA) antibody] was superior to sorafenib in the treatment of unresectable HCC. Patients in the atezolizumab plus bevacizumab group had significantly better 12-month OS than that in the sorafenib group (67.2% *vs*. 54.6%). Median PFS in the atezolizumab plus bevacizumab group was 6.8 months (95% CI: 5.7–8.3), whereas it was only 4.3 months in the sorafenib group (95% CI: 4.0–5.6). In this trial, 56.5% (186/329) of patients who received atezolizumab plus bevacizumab and 55.1% (86/156) of patients who received sorafenib had grade 3/4 adverse events (5).

In the COSMIC-312 trial (NCT03755791), the median PFS of cabozantinib plus atezolizumab and sorafenib monotherapy was 6.8 months (99% CI: 5.6–8.3) and 4.2 months (99% CI: 2.8–7.0) HR: 0.63, 99% CI: 0.44–0.91, p = 0.0012) for patients with advanced HCC; the median OS was 15.4 months (96% CI: 13.7–17.7) and 15.5 months (12.1–not estimable), respectively. Treatment-related adverse events occurred in 93% (399/429) of 429 patients in the cabozantinib plus atezolizumab group and 95% (178/188) of patients in the sorafenib group. Furthermore, 18% (78/433) of patients in the cabozantinib plus atezolizumab group and 8% (16/200) of patients in the sorafenib group experienced serious treatment-related adverse events (19). The HIMALAYA study showed that the OS for patients with unresectable HCC was dramatically improved with tremelimumab plus durvalumab compared to sorafenib HR: 0.78; 96% CI: 0.65–0.92; p = 0.0035). Moreover, 25.8%, 12.9%, and 36.9% of patients experienced grade 3/4 treatment-related adverse events in the tremelimumab plus durvalumab, durvalumab, and sorafenib groups, respectively (20). After a median follow-up of 10.0 months, the median PFS of sintilimab (a PD-1 inhibitor) plus IBI305 (a bevacizumab biosimilar) was 4.6 months (95% CI: 4.1–5.7) and sorafenib was 2.8 months (95% CI: 2.7–3.2) for patients with unresectable HCC in the ORIENT32 study (NCT03794440). In this study, 32% (123/384) of patients in the sintilimab plus IBI305 group and 19% (36/189) of patients in the sorafenib group experienced serious adverse events (21). Therefore, the paradigm for treating HCC has shifted as a result of the development of ICIs. ICIs are becoming more and more significant in the management of HCC.

## Underlying mechanisms of resistance to immune checkpoint inhibitors in hepatocellular carcinoma

**Figure 1:**
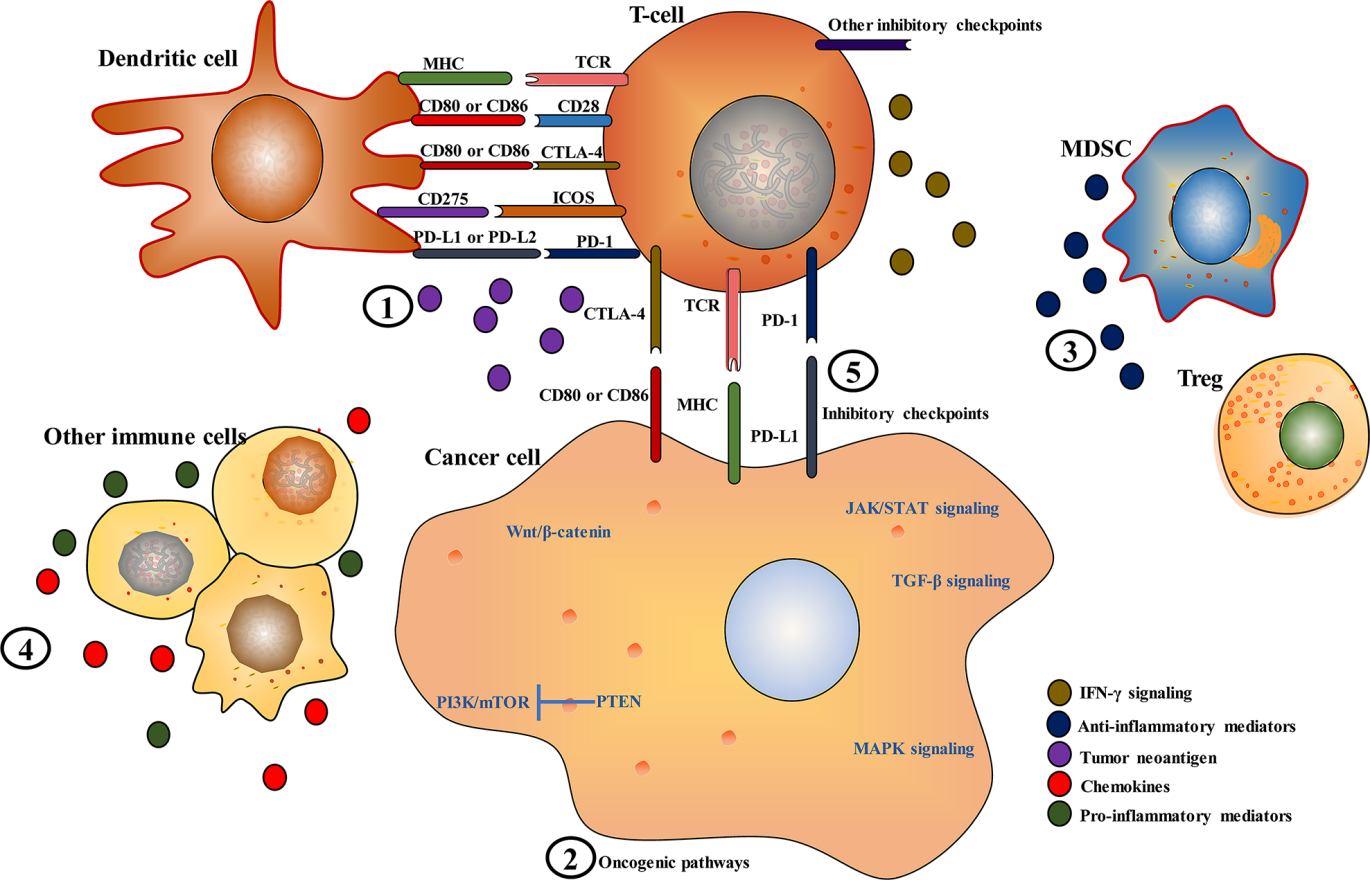
Key mechanisms of immune checkpoint inhibitor resistance in hepatocellular carcinoma. (1) Inadequate, absence or alterations in the presentation or processing of tumor neoantigens; (2) alterations in oncogenic pathways, such as phosphatidylinositol-3-kinase (PI3K)/mammalian target of rapamycin (mTOR), mitogen-activated protein kinase (MAPK), Janus kinase/signal transducer and activator of transcription (JAK/STAT),  Wnt/β-Catenin, and transforming growth factor beta (TGF-β) signaling pathways; (3 and 4) polarization toward an immunosuppressive microenvironment by reducing pro-inflammatory mediators and increasing anti-inflammatory mediators; (5) other novel immune checkpoint molecules, such as T-cell immunoglobulin and ITIM domain (TIGIT), lymphocyte activation gene-3 (LAG-3), T-cell immunoglobulin and mucin-domain containing-3 (TIM-3), B7 homolog 3 protein (B7-H3), B and T lymphocyte attenuator (BTLA), V-domain immunoglobulin suppressor of T cell activation (VISTA), and inducible T-cell costimulatory (ICOS).

A proportion of patients with HCC benefit from ICIs. However, primary (intrinsic) and acquired resistance are the primary clinical barriers to improving the outcome of patients with advanced HCC (9). Primary resistance occurs when there is no initial response to immunotherapy. Acquired resistance often develops disease progression after an initial response to immunotherapy. Mechanisms of primary and acquired resistance to immunotherapy somehow overlap (22). Primary resistance is primarily associated with the innate inability of the immune system to initiate an efficient immune response. Changes in epigenetic or translational processes may lead to adaptive changes in tumor cells and the TME, resulting in immunotherapy resistance. Acquired resistance to ICIs may occur in cancer cells that experienced clonal evolution and genetic alterations. Both tumor intrinsic and extrinsic mechanisms may cause primary or acquired resistance to ICIs. Understanding the underlying molecular mechanisms behind ICI resistance is necessary to optimize current treatment strategies or develop innovative ones. Intratumor heterogeneity is a significant contributor to the fatal result of cancer, treatment failure, and drug resistance. Intratumor heterogeneity offers a variety of genetic and epigenetic materials for Darwinian evolution. The evolving mutational landscape may affect immune surveillance and response to ICIs (23, 24). In tumors, the cancer immunity cycle involves cancer cell antigen release, cancer antigen presentation, priming and activation, trafficking of T cells to tumors, infiltration of T cells into tumors, recognition of cancer cells by T cells, and killing of cancer cells. Immunotherapy resistance is closely associated with the alterations in the cancer immunity cycle (25, 26). Therefore, decreased neoantigen expression, impaired antigen recognition, ineffective antigen presentation, insufficient priming and activation of tumor-specific T cells, poor trafficking of the activated effector T cells to tumors, decreased infiltration of the activated effector T cells into tumors, insufficient cancer cell recognition by T cells, inadequate expansion of T cells or lack of costimulation, the presence of T-cell inhibitory factors or other T-cell inhibitory immune cells in the TME can cause resistance to immunotherapy in tumors. Here, mechanisms that contribute to the resistance of ICIs in HCC are roughly summarized (**Figure 1**).

Tumor intrinsic mechanisms are closely associated with tumor cell alterations that affect neoantigen expression, antigen presentation, and expression of immune co-inhibitory signals, resulting in defective antigen recognition and decreased T-cell recruitment and activity in tumors. In HCC, alterations in several oncogenic pathways, such as Wnt/β-Catenin, mitogen-activated protein kinase (MAPK), phosphatidylinositol-3-kinase (PI3K)/protein kinase B (AKT)/mammalian target of rapamycin (mTOR), and transforming growth factor beta (TGF-β) pathways, can affect T-cell recruitment and function.

Activation of tumor-intrinsic β-catenin signaling leads to T-cell exclusion and resistance to ICI therapy (27). Immune classification investigations established an immune exclusion class characterized by Wnt/CTNNB1 mutations that accounts for about 30% of HCCs (28, 29). Strong associations between T-cell exclusion and *CTNNB1* mutations in HCC revealed that β-catenin activation might contribute to immune evasion and immunotherapy resistance (29, 30). In HCC cells, activation of β-catenin hampers the recruitment of CD103+ dendritic cells (DCs) and antigen-specific T cells, resulting in a diminished antitumor immune response. Chemokines, such as C-C Motif Chemokine Ligand 5 (CCL5), C-X-C Motif Chemokine Ligand 1 (CXCL1), and Chemokine (C-C motif) ligand 20 (CCL20), were significantly decreased in CTNNB1-mutant tumors. CCL5 overexpression in β-catenin-driven HCC cells enhanced the recruitment of antigen-specific CD8+ T cells and CD103+ DCs and restored immune surveillance. Numerous studies have exhibited that an aberrant WNT/β-catenin pathway is closely associated with resistance to anti-PD-(L)1 therapy in HCC (28, 31, 32). According to a recent study, two of three HCC patients with *CTNNB1* mutations did not respond to anti-PD-1 therapy (31). In another study, none of 10 HCC patients with *CTNNB1* or *AXIN1* alterations responded to the anti-PD-(L)1 agent, while 53% (9/17) of patients without WNT/β-catenin pathway alterations attained durable stable disease as the best response, indicating that aberrant WNT/β-catenin pathway alterations may enhance immune evasion and resistance to anti-PD-(L)1 therapy (28). Suppression of β‐catenin signaling in HCC may improve antitumor T-cell activation, resulting in the production of CD8+ effector T cells, promoting their penetration into the TME, and reducing CD8+ T-cell exhaustion after an initial response to anti‐PD‐1 treatment (33). Moreover, the Wnt/β-catenin signaling pathway activation causes M2 macrophage polarization in HCC cells, and Wnt ligands produced by macrophages further trigger the Wnt/β-catenin signaling pathway (34).

TGF-β signaling is involved in the regulation of immune cells, such as myeloid-derived suppressor cells (MDSCs), Dendritic cells (DCs), natural killer (NK) cells, and tumor-associated macrophages (TAMs), and plays a critical role in the proliferation, development, and differentiation of immune cells. TGF-β stimulates SMAD Family Member 2/3 (SMAD2/3) and interacts with interleukin (IL)-21 to enhance the maturation of CD4+ T cells into Th17 cells, which produce IL-17 and contribute to the development of HCC (35). TGF-β acts as a suppressor of the Th1 and Th2 lineages by suppressing T-bet and Sox4 expression, respectively, and facilitating the transformation of Th1 cells into Th2 cells (36). TGF-β collaborates with Activating Transcription Factor 1 (ATF1) to decrease interferon (IFN)-γ expression in CD8+ cytotoxic T cells, impairing its anticancer efficacy (37). TGF-β induces the development of M2-type macrophages, which inhibit the CD8+ T cell, NK cell, and DC activity and enhance the CD4+ Treg activity. TGF-β stimulates PD-1 expression in antigen-specific T cells *via* the SMAD3-dependent pathway (38). Elevated TGF-β signaling may lead to T-cell exhaustion through activation of PD-1 signaling, and TGF-β signaling suppression may improve antitumor immunity in HCC. The TGF-β signaling may block immunotherapy with anti-PD-(L)1 antibody *via* increasing PD-L1 expression. The TGF-β signaling may stimulate Treg expansion and disrupt immunotherapy with anti-CTLA-4 antibodies. In HCC, immune checkpoint molecules are modulated by TGF-β1-mediated epithelial–mesenchymal transition (EMT) (39). The TGF-β signature in HCC may be a promising indicator for recognizing the “exhausted” immune signature (40).

MAPK-RAP1A signaling is closely associated with tumor-infiltrating immune cells (TICs) in HCC (41). In HCC cells, the epidermal growth factor receptor (EGFR)‐P38 MAPK axis enhanced the aerobic glycolysis in HCC cells, which may stimulate tumor immunosuppression. The EGFR‐P38 MAPK axis can enhance PD‐L1 expression through miR‐675‐5p and suppress HLA‐I (HLA‐ABC) expression *via* glycolysis‐related enzyme hexokinase 2 (HK2) in HCC (42). IFN-γ enhanced CD274 transcriptional activity, and MAPK signaling elevated the stability of CD274 mRNA. Suppression of the MAPK pathway blocked the EGF and IFN-induced overexpression of PD-L1 in HCC cells (43). RAF proto-oncogene serine/threonine-protein kinase (RAF) dimers and ERK signaling activation induce immunosuppression through MAPK/Nuclear factor kappa B (NF-κB)-dependent PD-L1 expression in HCC (44).

Phosphatase and tensin homolog (*PTEN*) loss can lead to PI3K signaling pathway activation, resulting in decreased T-cell infiltration and increased immunosuppression (45). *PTEN*-deficient tumors may display impaired stimulation of the type I IFN and NF-κB pathways, contributing to tumor progression owing to the immunosuppressive TME (46). Endoplasmic reticulum (ER)-stressed HCC cells produce exosomes to increase PD-L1 expression in macrophages, which further suppress T-cell activity *via* an exosome miR-23a/PTEN/AKT pathway (47).

IFN-γ interacts with IFN-γ receptors (IFNGRs) and activates Janus kinase/signal transducer and activator of transcription (JAK/STAT) signaling pathway, which further promotes an IFN-stimulated gene (ISG) transcriptional pathway and modulates the immune response. Abnormalities in IFN-γ receptors or IFN-γ pathway-related genes such as *JAK1*, *JAK2*, *IFNGR1*, and *IFNGR2* may hamper tumor immune response and cause ICI resistance (48). IFN-γ-induced elevation of B7-H1 expression through the JAK/STAT1 pathway is responsible for adaptive immune resistance in HCC (49). PD-L1 expression induced by IFN-γ can be mediated by the IFN-γ/JAK/STAT1/IFN regulatory factor 1 (IRF1) pathway in HCC cells. MYC (MYC proto-oncogene, bHLH transcription factor) suppression increased STAT1 expression, a crucial component of the IFN-γ signaling pathway, increasing PD-L1 expression induced by IFN-γ in HCC cells (50). The aberrant activation of the IL-10/JAK1/STAT5/Foxp3 pathway may facilitate the accumulation of Tregs in the TME (51).

Other tumor cell-intrinsic mechanisms are closely associated with the expression of co-inhibitory signals, inadequate antigen expression, and impaired antigen processing and presentation. Tumor-specific neoantigens are the repertoires of peptides that bind to the MHC to form a stable complex for T-cell recognition, thereby eliciting anticancer T-cell responses. Tumor cells may evade immune surveillance through insufficient antigen and genetic and epigenetic changes in antigens (52). The β2-microglobulin (B2M) is a component of MHC Class I molecules, and homozygous B2M deficiency causes inadequate antigen presentation, hindering the recognition of CD8+ T cells (8). The function of the MHC to present neoantigens was impeded by loss of heterozygosity (LOH) of human leukocyte antigen (HLA) alleles, which was detected in 17% of multifocal HCC and associated with immune escape (53).

Tumor extrinsic mechanisms include the status of the host immune homeostasis and the features of the TME, which may affect T-cell priming and activation, antigen recognition, immune cell recruitment and activity, and co-inhibitory or costimulatory signals. The TME contains TAMs, MDSCs, and Tregs that exert immunosuppressive effects. These immunosuppressive immune cells interact with immunosuppressive cytokines, such as Indoleamine 2,3-dioxygenase (IDO), TGF-β, adenosine, IL-10, and CD73, to decrease antigen presentation and cytotoxic T-cell activity (54). Various immune checkpoint molecules such as PD-1, lymphocyte activation gene-3 (LAG-3), T-cell immunoglobulin and mucin-domain containing-3 (TIM-3), CTLA-4, T-cell immunoglobulin and ITIM domain (TIGIT), and V-domain immunoglobulin suppressor of T-cell activation (VISTA) exist within the TME. Immune cells and APCs in the TME may induce the expression of these immune checkpoint molecules, promoting T-cell exhaustion and hence decreasing the response to ICIs (55).

Tumor-associated neutrophils (TANs) cause CD8 T-cell apoptosis through the tumor necrosis factor (TNF)-α pathway, contributing to the immunosuppressive response. TANs and granulocytic myeloid-derived suppressor cells (G-MDSCs) inhibit the proliferation, activation, and antitumor activity of CD8 T cells. Moreover, TANs can inhibit the proliferation of T cells by releasing arginase 1 (ARG1) and regulating PD-L1/PD-1 signaling and induce an immunosuppressive response (56, 57).

Tregs can block immunological responses and sustain homeostasis and self-tolerance. Furthermore, Tregs can decrease T-cell proliferation and cytokine secretion and suppress autoimmunity (58). Treg enrichment was found in primary HCC, resulting in an immunosuppressive setting, while Treg exclusion and CD8+ T cell enrichment were detected in early-recurrent HCC (59). Infiltrating Tregs and exhausted CD8+ T cells are clonally enriched in HCC. Layilin (LAYN) is a signature gene for tumor-specific Tregs and exhausted CD8+ T cells, and LAYN overexpression in CD8+ T cells suppressed the production of IFN-γ, suggesting that LAYN had an inhibitory effect on CD8+ T cells. T cells in the TME are prone to exhaustion or Treg suppression, blocking CD8 cells from inducing T cell-mediated tumor cell killing (60).

The primary mechanisms by which MDSCs suppress immune responses in the TME include encouraging Treg differentiation and expansion, blocking DC, NK, and macrophage polarization to the M2 phenotype, depriving essential amino acids of T cells, and causing oxidative stress, indicating that MDSCs participate in multiple immunosuppressive pathways and are viable immunotherapeutic targets for HCC (61). MDSCs aggregate in the TME and act as pro-inflammatory mediators, inhibiting T‐cell activities and contributing to immune evasion. Triggered MDSCs produce cytokines and enzymes that suppress NK cells and T cells and stimulate Tregs. Hepatic receptor-interacting protein kinase 3 (RIP3) defect stimulates chemokine (CXC motif) ligand 1 (CXCL1)/chemokine (CXC motif) receptor 2 (CXCR2)–induced MDSC recruitment and decreases IFN+ CD8+ T-cell infiltration, thereby facilitating immune escape and HCC progression (62). MDSCs suppress hepatic NK cell function through the TGF-β pathway and promote Treg expansion (40).

TAMs typically recruit monocytes from the periphery through the action of chemokines and subsequently deposit them in tumor tissue. Tissue-resident macrophages move to hypoxic or necrotic regions of tumors where they are stimulated to undergo TAM transformation. TAMs evolved from monocytes into functional macrophages and acquired a range of immunosuppressive functions to maintain the TME. TAMs produce cytokines and chemokines, which stimulate tumor growth and suppress antitumor immunity (63, 64). M2 TAMs facilitate HCC progression *via* producing protumor and proangiogenic factors and inhibiting tumor-infiltrating T cell activation, while M1 TAMs secrete IL1b, reactive oxygen species (ROS), and other pro-inflammatory cytokines to inhibit tumor progression. TAMs are the predominant immunosuppressive component in the TME of HCC (65). Receptor-interacting protein kinase 3 (RIPK3) deficiency accelerated TAM polarization toward an M2 phenotype and facilitated the immunosuppressive function of TAMs (66).

Immunosuppressive cells, such as TAMs, TANs, MDSCs, tumor-associated DCs (tDCs), and Tregs, are important contributors to immune resistance and can also affect the therapeutic potential of ICIs (67, 68). Numerous mechanisms of immunotherapeutic resistance have been identified and well-described, and new ones are constantly being found. To aid in therapeutic decision-making, further knowledge of resistance mechanisms is required.

## Potential strategies to overcome immune checkpoint inhibitor resistance in hepatocellular carcinoma

**Figure 2:**
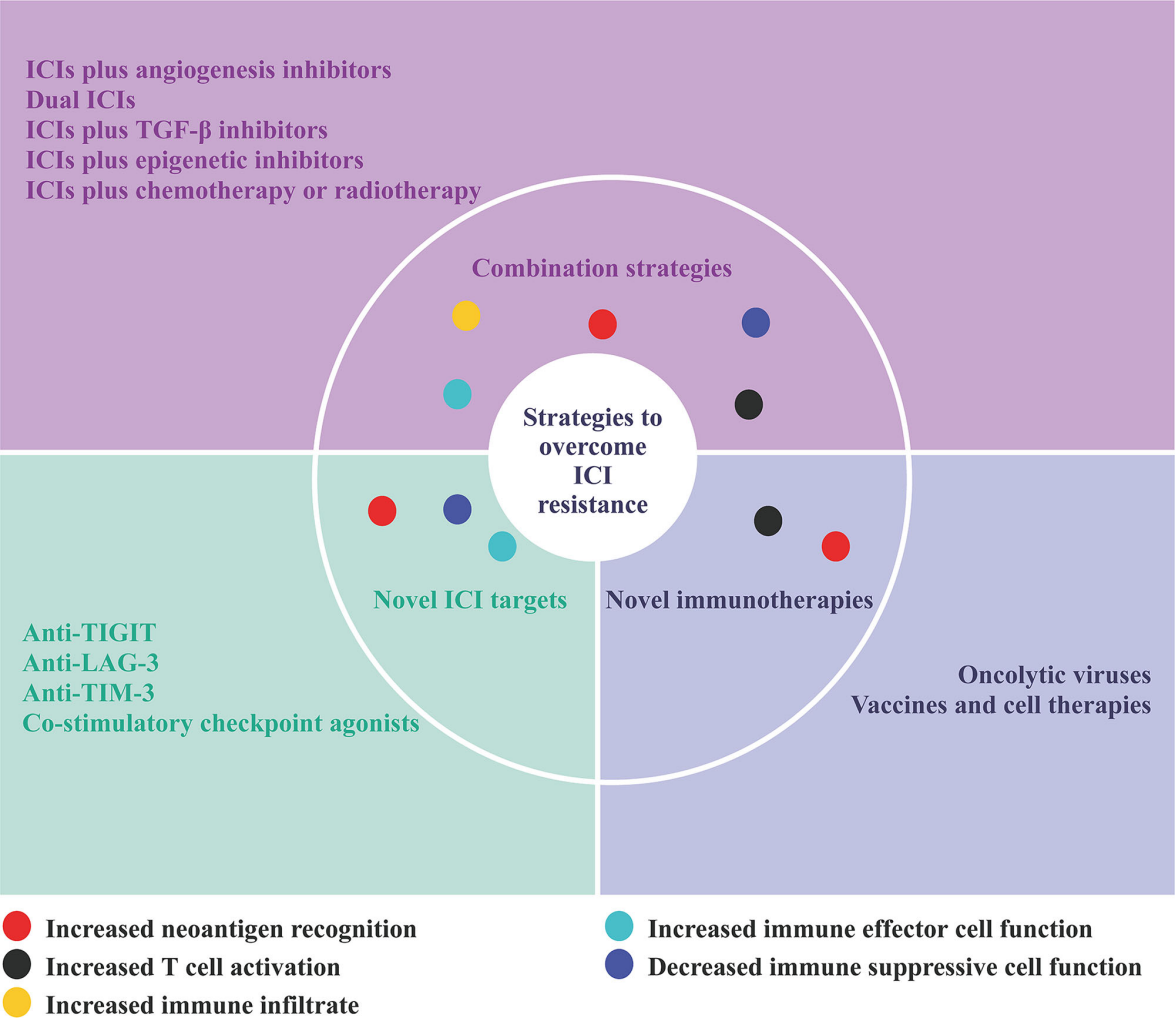
Strategies to overcome ICI resistance.

As the mechanisms of ICI resistance are understood, several therapeutic strategies have been developed to overcome ICI resistance. Current efforts to overcome ICI resistance for advanced HCC mainly focus on the following aspects: combination strategies involving ICIs, novel ICI targets, and novel immunotherapies (**Figure 2**) (69).

### Combination strategies

#### Immune checkpoint inhibitors plus angiogenesis inhibitors

Numerous immunosuppressive mechanisms involved in the recurrence of HCC are regulated by VEGF or/and immune checkpoints. The combination of ICIs and antiangiogenic agents has been investigated in several clinical studies, and the results showed that dual blockade of ICIs and VEGF can improve anticancer immunity. The combination of ICIs and antiangiogenic agents may exert synergistic therapeutic effects through multiple mechanisms, such as vascular normalization, antitumor immune cell subset activation, and inhibition of tumor-promoting immune cells. Anti-PD-1/PD-L1 treatments may enhance recruitment or activation of DCs, effector CD8+ T cells, and NK cells and induce an antitumor M1 macrophage phenotype. Anti-VEGF treatments may decrease MDSC and Treg infiltration and activity and diminish the polarization of M2 macrophages. Suppression of DC maturation, inadequate antigen presentation, suppression of T-cell responses *via* upregulating PD-L1, PD-L2, and immunosuppressive molecules (IL-6, IDO-1, and IL-10), initiation of Tregs, and accumulation of MDSCs are some of the mechanisms by which VEGF has a significant impact on cancer immunity (69). In a phase III trial (IMbrave150), atezolizumab (PD-L1 antibody) plus bevacizumab (antiangiogenic agent) significantly improved overall and PFS compared to sorafenib, thus becoming the new standard of first-line treatment for advanced HCC (5, 70). Other combinations of ICIs and antiangiogenic agents, including durvalumab (an anti-PD-L1 agent) plus bevacizumab (EMERALD-1), pembrolizumab (an anti-PD-1 agent) plus lenvatinib (LEAP-012; LEAP-002), regorafenib plus nivolumab (an anti-PD-1 agent) (RENOTACE), atezolizumab (an anti-PD-L1 agent) plus cabozantinib (COSMIC-312) (19), camrelizumab (an anti-PD-1 agent) plus apatinib (NCT03764293), CS1003 (an anti-PD-1 agent) plus lenvatinib (NCT04194775), sintilimab (an anti-PD-1 agent) plus IBI305 (ORIENT-32) (21) and atezolizumab plus lenvatinib or sorafenib (IMbrave251), are under clinical investigation in HCC (69).

#### Dual immune checkpoint inhibitors

Dual ICIs are another promising strategy in HCC. The CheckMate 040 study presented the efficacy and safety of nivolumab (an anti-PD-1 inhibitor) in combination with ipilimumab (a CTLA-4 inhibitor) in advanced HCC. The U.S. Food and Drug Administration (FDA) has approved this strategy for the treatment of patients with advanced HCC (4). Other combinations of dual ICIs, including durvalumab (an anti-PD-L1 agent) plus tremelimumab (a CTLA-4 inhibitor) (EMERALD-2; HIMALAYA), are under clinical investigation in HCC (69). Other dual ICI strategies, including an anti-PD-1/PD-L1 agent plus an anti-TIM-3/anti-LAG-3/anti-TIGIT agent, are currently in clinical trials. Agonists targeting costimulatory pathways such as OX40 (CD134)/OX40L (CD252), CD40/CD40L, Glucocorticoid-induced tumor necrosis factor receptor-related protein (GITR)/GITR ligand (GITRL), CD27/CD70, inducible T-cell costimulatory (ICOS)/ICOS ligand (ICOSL), 4-1BB (CD137, tumor necrosis factor receptor superfamily 9)/4-1BB ligand (4-1BBL) can stimulate T-cell activity and activate antitumor immunity. These combination strategies showed great potential in preclinical studies, and more research on patients with advanced solid tumors is warranted. Numerous clinical trials that combine costimulatory molecule agonists and α-PD-1/PD-L1 are ongoing in advanced solid tumors (71).

#### Immune checkpoint inhibitors plus TGF-β inhibitors

TGF-β, an immunosuppressive cytokine, inhibits effector T-cell activity and infiltration and increases Treg activity. Preclinical studies have indicated that inhibiting TGF-β signaling makes tumor cells more susceptible to ICIs. Targeting the TGF-β pathway with galunisertib (an anti-TGF-β agent) can increase the antitumor activity of anti-PD-(L)1 agents in a preclinical study (72). In a phase II study (NCT02423343), galunisertib (LY2157299) was evaluated with nivolumab in patients with advanced refractory solid tumors, including HCC. In addition, the TGF-β signature may serve as a potential biomarker for individualized immunotherapy in patients with HCC (40).

#### Immune checkpoint inhibitors plus epigenetic inhibitors

Epigenetic modulation may alleviate resistance to ICIs in HCC tumors with low immunogenicity and inadequate antigen presentation. DNA methyltransferase inhibitor therapy reverses MHC class I epigenetic inhibition and promotes antigen presentation, immunogenicity, and tumor immune targeting. In a humanized mouse model of immune deficiency, histone deacetylase 8 (HDAC8) inhibition elevated global and enhancer acetylation of H3K27, allowing HCC cells to generate T cell-trafficking chemokines and alleviating T-cell exclusion. HDAC8 inhibition enhanced tumor-infiltrating CD8+ T cells, improving the efficacy of the anti-PD-L1 agent in HCC (73). HDAC6 inhibits the pathogenicity of IL-17-producing helper T (TH 17) cells and the antitumor immune function *via* modulating forkhead box protein O1 (FoxO1). HDAC6-depleted T cells induce PD-1-PD-L1 expression to generate a powerful synergistic impact that makes advanced HCC more susceptible to ICIs (74). SGI-110 (a DNA methyltransferase inhibitor) inhibits both DNA methyltransferases and the PRC2 (polycomb repressive complex 2) complex in HCC. It can stimulate endogenous retrovirus (ERV) to activate the immune response pathway of cancer cells, offering a foundation for the combination therapy of DNA methyltransferase inhibitors and ICIs (75).

#### Immune checkpoint inhibitors plus chemotherapy or radiotherapy

Radiotherapy or chemotherapy can not only kill cancer cells but also modulate immunity. Numerous preclinical studies have demonstrated that the combination of radiotherapy or chemotherapy and ICIs is promising for multiple cancers, and several related clinical trials are currently underway. These combination strategies may improve the microenvironment for immune cells to interact with the tumor antigenic environment. However, more clinical evidence is needed to confirm whether radiotherapy or chemotherapy can enhance the immunotherapy effect of ICIs in HCC (76).

### Novel immune checkpoint inhibitor targets

Targeting alternative immune checkpoints is one strategy for overcoming ICI resistance. Immune checkpoints such as TIGIT, LAG-3, TIM-3, B7 homolog 3 protein (B7-H3), B and T lymphocyte attenuator (BTLA), VISTA, and ICOS are viable and attractive targets for solid tumor treatment. Relevant clinical studies are now underway (**Table 2**) (77, 78).

**Table 2:** Novel immune checkpoint inhibitors in HCC.

Drug	Target	NCT number	Phase	Settings	Treatment arms
Relatlimab	LAG-3	NCT04658147	I	Hepatocellular carcinoma	Nivolumab; Nivolumab + Relatlimab
SRF388	LAG-3	NCT04374877	I	Advanced solid tumor	SRF388; SRF388 + Pembrolizumab
Relatlimab	LAG-3	NCT04567615	II	Liver cancer	Nivolumab; Nivolumab + Relatlimab
INCAGN02385	LAG-3	NCT03538028	I	Advanced solid tumor	INCAGN02385
Relatlimab	LAG-3	NCT02465060	II	Advanced solid tumor	Relatlimab
Pavunalimab	CTLA-4 and LAG-3	NCT03849469	I	Advanced solid tumor	Pavunalimab; Pavunalimab + Pembrolizumab
Cobolimab	TIM-3	NCT03680508	II	Liver cancer	Dostarlimab + Cobolimab
INCAGN02390	TIM-3	NCT03652077	I	Advanced solid tumor	INCAGN02390
KY1044	ICOS	NCT03829501	I and II	Advanced solid tumor	KY1044; KY1044 + Atezolizumab

LAG-3 (CD223), a membrane receptor, is mainly expressed on activated T cells and NK cells. When LAG-3 binds with MHC class II, it suppresses the activity of T cells. Blocking the interaction between LAG-3 and MCH II with LAG-3 inhibitors induces immune activation and antitumor activity. In a preclinical study, LAG-3 and PD-1 can be coexpressed on T cells, and LAG-3 and PD-1/PD-L1 may synergistically modulate T-cell activity to facilitate immune escape, suggesting that the combination of anti-PD-(L)1 antibody and anti-LAG-3 antibody may be a feasible strategy, especially for patients who are resistant to anti-PD-(L)1 inhibitor therapy (79). In the phase III RELATIVITY-047 (NCT03470922) study, the median PFS of patients with advanced melanoma treated with relatlimab (an anti-LAG-3 antibody) plus nivolumab (an anti-PD-1 antibody) was substantially longer than that of patients treated with nivolumab with a median follow-up duration of 13.2 months [10.1 months *vs*. 4.6 months; HR: 0.75 (95% CI: 0.62–0.92); p = 0.006]. Grade 3/4 treatment-related adverse events were reported more commonly in the relatlimab-nivolumab group than in the nivolumab group (18.9% *vs*. 9.7%) (80). A phase I trial (NCT04658147) is currently recruiting patients to evaluate the feasibility and efficacy of nivolumab with or without relatlimab in the treatment of HCC. A phase II trial (NCT04567615) is currently enrolling patients to assess the safety and efficacy of nivolumab plus relatlimab in treating advanced HCC patients. Pavunalimab (XmAb22841) is a bispecific antibody that targets CTLA-4 and LAG-3 and may contribute to an improved antitumor immune response. A phase I multiple-dose study (NCT03849469) is currently enrolling patients to assess the safety and tolerability of pavunalimab monotherapy and in combination with pembrolizumab in patients with advanced solid tumors. Moreover, preclinical studies demonstrate that IBI323, a bispecific antibody against PD-L1/LAG-3, improves tumor-specific immunity, suggesting that dual-blockade bispecific antibodies targeting PD-L1 and LAG-3 may represent a viable treatment option (81).

TIM-3 acts as a negative modulator of T lymphocytes. When it interacts with its ligands, it can induce T-cell exhaustion and the production of MDSCs in the TME and promote tumor growth. TIM-3 inhibition lowers MDSCs, enhances T-cell proliferation and cytokine secretion, and improves antitumor efficacy. The presence of cytokines such as IL-4, TGF-β, and IL-6 in the TME induces the expression of TIM-3 in HCC cells. Tumor cell-intrinsic TIM-3 stimulates NF-κB phosphorylation, which increases IL-6 production and STAT3 phosphorylation (82). TIM-3 facilitates the development of HCC *via* stimulating TGF-β-mediated alternative macrophage activation, suggesting that TIM-3 interference may have significant therapeutic implications for HCC (83). Several TIM-3 inhibitors are being evaluated in clinical trials, including LY3321367, Sym023, TSR-022, and BGB-A425. Moreover, clinical trials evaluating the safety, tolerability, and efficacy of TIM-3/PD-(L)1 bispecific antibodies such as RO7121661 and LY3415244 are ongoing (84).

TIGIT, an immune checkpoint, is mainly expressed on T cells and NK cells. By interacting with ligands with its ligands CD155, PVRL3, and CD112, TIGIT blocks NK cell-mediated tumor killing, activates immunosuppressive DCs, decreases CD8 T-cell initiation and differentiation, and inhibits CD8 T cell-mediated tumor killing, thus causing immunosuppression. Several anti-TIGIT agents have been investigated in clinical trials. TIGIT inhibitors such as ociperlimab, tiragolumab, and BMS-986207 may be effective against solid tumors alone or with a PD-(L)1 inhibitor. Moreover, bispecific antibodies against PD-(L)1/TIGIT, such as HLX301 and AZD2936, have significant therapeutic potential in the future (85).

Several checkpoint molecules show positive immunoregulatory activities in the setting of cancer. Immune costimulator (ICOS) is a costimulatory molecule secreted on T cells, which stimulates CD8+ T-cell and Treg activity. ICOS agonist monoclonal antibodies such as JTX-2011, feladilimab, and BMS-986226 are being explored as single agents or with anti-PD-(L)1/anti-CTLA-4 therapies (86). In addition, glucocorticoid-induced TNFR-related gene (GITR) or OX40, belonging to the TNF receptor superfamily, is being evaluated as stimulatory factors. GITR is mainly expressed on effector T cells, Tregs, and NK cells, where it interacts with its ligand GITRL to perform positive immunoregulatory effects. OX40 can promote T-cell expansion and modulate T helper activation (87). Clinical trials for GITR agonist antibodies (BMS-986156 and TRX518) and OX40 agonist antibodies (MEDI6469 and PF-04518600) are undergoing in solid tumors.

### Novel immunotherapies

Developing novel strategies for ICI resistance management is attracting growing interest. A growing body of evidence demonstrated the significance of oncolytic viruses, gut and tumor microbiome, vaccines, and cell therapies in tumor immunity. For example, oncolytic viruses destroy tumor cells through a variety of mechanisms, including inducing cytotoxicity, lysis, and activation of antitumor immune responses. Oncolytic viruses have immune-stimulating effects and can transform non-inflammatory microenvironments into inflammatory microenvironments, and their combination with ICI strategies may improve therapeutic outcomes (88). A phase I/II study (NCT02509507) evaluating talimogene laherparepvec (a modified oncolytic virus) plus pembrolizumab in patients with HCC or other solid tumors with liver metastases is ongoing. Based on a phase II study (NCT00554372), JX-594 (an oncolytic and immunotherapeutic vaccinia virus) can kill HCC cells through viral oncolysis and immunity, and it shows promise as a treatment strategy for advanced HCC, and further clinical studies are warranted (89). A better understanding of the underlying mechanisms of these factors is a prerequisite for the application of these emerging strategies.

## Prospects and conclusions

ICIs have revolutionized advanced HCC treatment. However, ICIs can elicit a durable antitumor response in only a subset of HCC, and several mechanisms can lead to primary or acquired resistance to ICIs. Anti-PD-(L)1 ICIs plus anti-VEGFA agents provide patients with a favorable objective response rate. The approval of atezolizumab plus bevacizumab is a new milestone in treating advanced HCC. Treatment strategies after the resistance to anti-PD-(L)1 ICIs in combination with anti-VEGFA agents remain to be determined. Whether patients who once benefited from atezolizumab plus bevacizumab might respond to other ICI-based combination strategies remains unclear. Identification of patients at risk of ICI resistance and timely change of treatment strategy are currently feasible options to overcome ICI resistance. The high burden of single-nucleotide variants and small insertion and deletion can induce increased neoantigen abundance and enhanced mutant binding specificity. Tumor cells with inherently low TMB are more likely to be deficient in neoantigens and are more susceptible to ICI resistance. Decreased production of MHC-I and β2-microglobulin can affect how tumor antigens are processed or presented and cause ICI resistance. Additionally, epigenetic alterations can affect how antigens are processed and presented, alter how cytokines are produced, and result in ICI resistance. Moreover, the abnormal IFN-γ pathway is a significant factor in ICI resistance. Developing combinatorial biomarkers to predict response to ICIs is an urgent need. In addition to the currently widely used immunotherapy markers (PD-L1 expression, TMB, and MMR/MSI), tumor-intrinsic biomarkers (neoantigens, WNT/β-catenin, DNA damage pathways, IFN signaling mutations, AT-rich interactive domain-containing protein 1A (ARID1A) mutation, PTEN loss) and immune-specific biomarkers (T cells, IFN-γ signature, Teff/Treg ratio, CD103+ DCs, tumor lysis syndrome (TLS), B-cell signature, CXCL13, M1 and M2 cells) are worthy of further exploration. Biomarker-based treatments make treatment more precise, and identifying the molecular alterations that cause ICI resistance may provide a new standard of therapy.

Further research is needed to elucidate the mechanisms of resistance to ICIs in patients with HCC. In particular, the influence of different immune cell subsets and signaling pathways on ICI treatment response requires further explanation. In addition, the relationship between gene signatures, immune classes and specific mutations, and treatment response or resistance needs to be further explored. Closer integration of cancer immunotherapy with fields including cancer biology, computational biology, and epigenetics will contribute to a better understanding of the mechanisms of ICI resistance. Further advancement of ICIs is required in combination with other therapies such as targeted therapy, chemotherapy, radiotherapy, or other immunotherapies such as novel ICIs, and chimeric antigen receptor T cells. It is also worth exploring how to select the appropriate combination therapy more accurately. The future of ICIs is promising, and they still have the potential to further improve outcomes in HCC.

## Author contributions

QX and CZ conceived of and designed the work. PZ, YW, WM drafted, and revised the manuscript. All authors contributed to the article and approved the submitted version.

## Funding

This present study was supported in part by the National Natural Science Foundation of China (grant numbers: 81660755), and the Science and Technology Project of Shenzhen of China (grant numbers: JCYJ2021032414261403 and JCYJ20190808162605484).

## Conflict of interest

The authors declare that the research was conducted in the absence of any commercial or financial relationships that could be construed as a potential conflict of interest.

## Publisher’s note

All claims expressed in this article are solely those of the authors and do not necessarily represent those of their affiliated organizations, or those of the publisher, the editors and the reviewers. Any product that may be evaluated in this article, or claim that may be made by its manufacturer, is not guaranteed or endorsed by the publisher.
